# A slow-releasing donor of hydrogen sulfide inhibits neuronal cell death via anti-PANoptosis in rats with spinal cord ischemia‒reperfusion injury

**DOI:** 10.1186/s12964-023-01457-x

**Published:** 2024-01-12

**Authors:** Lei Xie, Hang Wu, Qiuping He, Weipeng Shi, Jing Zhang, Xiao Xiao, Tengbo Yu

**Affiliations:** 1 Department of Orthopedic Surgery, Qingdao Municipal Hospital, Qingdao University, Qingdao, China; 2 Institute of Sports Medicine and Health, Qingdao University, Qingdao, China; 3 Department of Orthopedics, The Affiliated Hospital of Qingdao University, Qingdao University, Qingdao, China; 4 Central Laboratories, Qingdao Municipal Hospital, University of Health and Rehabilitation Sciences, Qingdao, China; 5 Department of Orthopedic Surgery, Qingdao Municipal Hospital, University of Health and Rehabilitation Sciences, Qingdao, China

**Keywords:** PANoptosis, Hydrogen sulfide, Inflammation, Microglia/macrophage, Spinal cord ischemia‒reperfusion injury

## Abstract

**Background:**

Spinal cord ischemia‒reperfusion injury (SCIRI) can lead to paraplegia, which leads to permanent motor function loss. It is a disastrous complication of surgery and causes tremendous socioeconomic burden. However, effective treatments for SCIRI are still lacking. PANoptosis consists of three kinds of programmed cell death, pyroptosis, apoptosis, and necroptosis, and may contribute to ischemia‒reperfusion-induced neuron death. Previous studies have demonstrated that hydrogen sulfide (H_2_S) exerts a neuroprotective effect in many neurodegenerative diseases. However, whether H_2_S is anti-PANoptosis and neuroprotective in the progression of acute SCIRI remains unclear. Thus, in this study we aimed to explore the role of H_2_S in SCIRI and its underlying mechanisms.

**Methods:**

Measurements of lower limb function, neuronal activity, microglia/macrophage function histopathological examinations, and biochemical levels were performed to examine the efficacy of H_2_S and to further demonstrate the mechanism and treatment of SCIRI.

**Results:**

The results showed that GYY4137 (a slow-releasing H_2_S donor) treatment attenuated the loss of Nissl bodies after SCIRI and improved the BBB score. Additionally, the number of TUNEL-positive and cleaved caspase-3-positive cells was decreased, and the upregulation of expression of cleaved caspase-8, cleaved caspase-3, Bax, and Bad and downregulation of Bcl-2 expression were reversed after GYY4137 administration. Meanwhile, both the expression and activation of p-MLKL, p-RIP1, and p-RIP3, along with the number of PI-positive and RIP3-positive neurons, were decreased in GYY4137-treated rats. Furthermore, GYY4137 administration reduced the expression of NLRP3, cleaved caspase-1 and cleaved GSDMD, decreased the colocalization NeuN/NLRP3 and Iba1/interleukin-1β-expressing cells, and inhibited proinflammatory factors and microglia/macrophage polarization.

**Conclusions:**

H_2_S ameliorated spinal cord neuron loss, prevented motor dysfunction after SCIRI, and exerted a neuroprotective effect via the inhibition of PANoptosis and overactivated microglia-mediated neuroinflammation in SCIRI.

**Supplementary Information:**

The online version contains supplementary material available at 10.1186/s12964-023-01457-x.

## Background

Spinal cord ischemia‒reperfusion injury (SCIRI) refers to a disastrous complication of thoracoabdominal aneurysm surgery and endovascular aortic repair surgery that can also occur with spinal trauma, degeneration, or tumors, which leads to devastating sensory and motor dysfunction [1, 2]. Despite advances in technology and operative skill, approximately 5–18% of patients still experience SCIRI [3]. Paraplegia places a tremendous financial and psychological burden not only on the patients and their families but also on society [4]. To date, including cerebrospinal fluid drainage [5], reattachment of segmental arteries [6], and administration of pharmaceuticals drugs (steroids, oxygen-derived free radical scavengers, and vasodilators) [7, 8], are unsatisfactory in preventing the progression of paraplegia [9]. Therefore, it is particularly important to explore the molecular and cellular mechanisms of SCIRI and look for effective, feasible and applicable treatments.

Programmed cell death (PCD) plays a key role in organismal evolution and immune response [10]. Panoptosis (pyroptosis, apoptosis, and necroptosis) is a new form of PCDs that is widely involved in various pathological processes [11, 12], such as cancer [13], cerebral ischemia [14], and retinal ischemia‒reperfusion injury [15]. Nucleotide-binding oligomerization domain (NOD)-like receptor pyrin domain-containing 3 (NLRP3)-dependent pyroptosis, caspase-dependent apoptosis and receptor-interacting protein (RIP)-dependent necroptosis are three types of PANoptosome components [13]. NLRP3-dependent pyroptosis has been shown to participate in neuron death in SCIRI [16]. Apoptosis also induces neuronal death and motor dysfunction [17]. In addition, much evidence implies that necrosis contributes to ischemia‒reperfusion-induced neuron degeneration and that RIP1 inhibitors can promote neuron survival [18]. Thus, these results suggest that PANoptosis may be involved in neuronal death caused by ischemia‒reperfusion [14, 15, 19]. However, the underlying pathophysiological mechanisms of PANoptosis in SCIRI have not been fully elucidated and deserve further investigation.

Hydrogen sulfide (H_2_S) is an endogenously generated gasotransmitter along with nitric oxide and carbon monoxide that is produced by enzymes including cystathionine γ-lyase, cystathionine β-synthase, and 3-mercaptopyruvate sulfurtransferase [20]. Previously considered a hazardous gas, extensive studies have demonstrated that H_2_S plays various physiological and pathological roles in biological systems, such as those related to neurophysiology, cardiovascular disease and endocrine regulation, highlighting many potential applications of H_2_S donors in therapy [21]. Recently, the study of H_2_S in chemistry, biochemistry, biology, and medicinal disciplines has become a popular area of research [22]. In many signal transduction pathways, H_2_S is a key regulator. For example, it can induce persulfidation of the parkin protein and thereby prevent Parkinson’s disease [23]. In addition, pathological processes involved in cardiovascular diseases, kidney diseases, and gastrointestinal diseases have been found to be closely related to H_2_S [24–26]. We previously reported that exogenous H_2_S exerts protective effects on rats with SCIRI [27]. Its administration neuron survival by reducing the number of apoptotic neurons. In addition, it has a neuroprotective effect through its antioxidative and anti-inflammatory effects [28]. The relationship between H_2_S and PANoptosis has not been reported. However, clarifying the mechanism underlying the effect of H_2_S is crucial for the development of H_2_S-based therapy for the treatment of SCIRI and for translation from “bench to bedside”. Herein, we sought to investigate whether H_2_S ameliorated SCIRI and was associated with anti-PANoptosis.

Here, our findings suggest that the efficacy of H_2_S could mitigate the PANoptosis induced by SCIRI in rats. Specifically, we used a combination of measurements of lower limb function, neuronal activity, inflammatory factors, and microglia/macrophage function to examine the efficacy of H_2_S and to further demonstrate the mechanism and treatment of SCIRI. The results of our studies not only reveal the protective properties of H_2_S at a proper dosage in the SCIRI rat model, but also demonstrate that H_2_S may ameliorate the consequences of SCIRI by decreasing pyroptosis, apoptosis and necroptosis of neurons, polarization of microglia/macrophages, and inflammation.

## Materials and methods

### Animals

Male Sprague‒Dawley rats (200–220 g weight), purchased from the Animal Laboratory of Beijing Charles River Corporation (Beijing, China), were used in this study. The animals were housed with ad libitum access to water and food in an air-conditioned room with a 12-hours light/dark cycle at 25 °C and 50% relative humidity and were acclimated to their surroundings for one week prior to experiments.

### Ethics statement

All animal experiments and procedures were reviewed and approved in accordance with the protocol approved by the Qingdao University Laboratory Animal Welfare Ethics Committee (No.20230420SD20230612079) and in accordance with the Ministry of Science and Technology of the People’s Republic of China Animal Care guidelines. All surgeries were performed under anesthesia, and all efforts were made to minimize animal suffering.

### Spinal cord ischemia‒reperfusion injury (SCIRI) model

The SCIRI model was generated using a modification of a previously reported method [17]. Briefly, all rats were neurologically intact before the experiment and were anesthetized. The rats in the SCIRI group, the abdominal aorta was blocked below the right renal artery near the heart using a 50 g aneurysm clip for 60 min. All rats were placed in a box at 28 °C to recover from anesthesia and were subsequently placed in separate cages with ad libitum access to food and water.

### Drug preparation and procedures

Morpholin-4-ium 4 methoxyphenyl(morpholino) phosphinodithioate (GYY4137) was purchased from MedChemExpress (HY-107632), dissolved in dimethyl sulfoxide to yield a stock solution of 100 mg/ml and further diluted in phosphate-buffered saline (PBS) to generate the final dose before intraperitoneal injection.

**Fig. 1:**
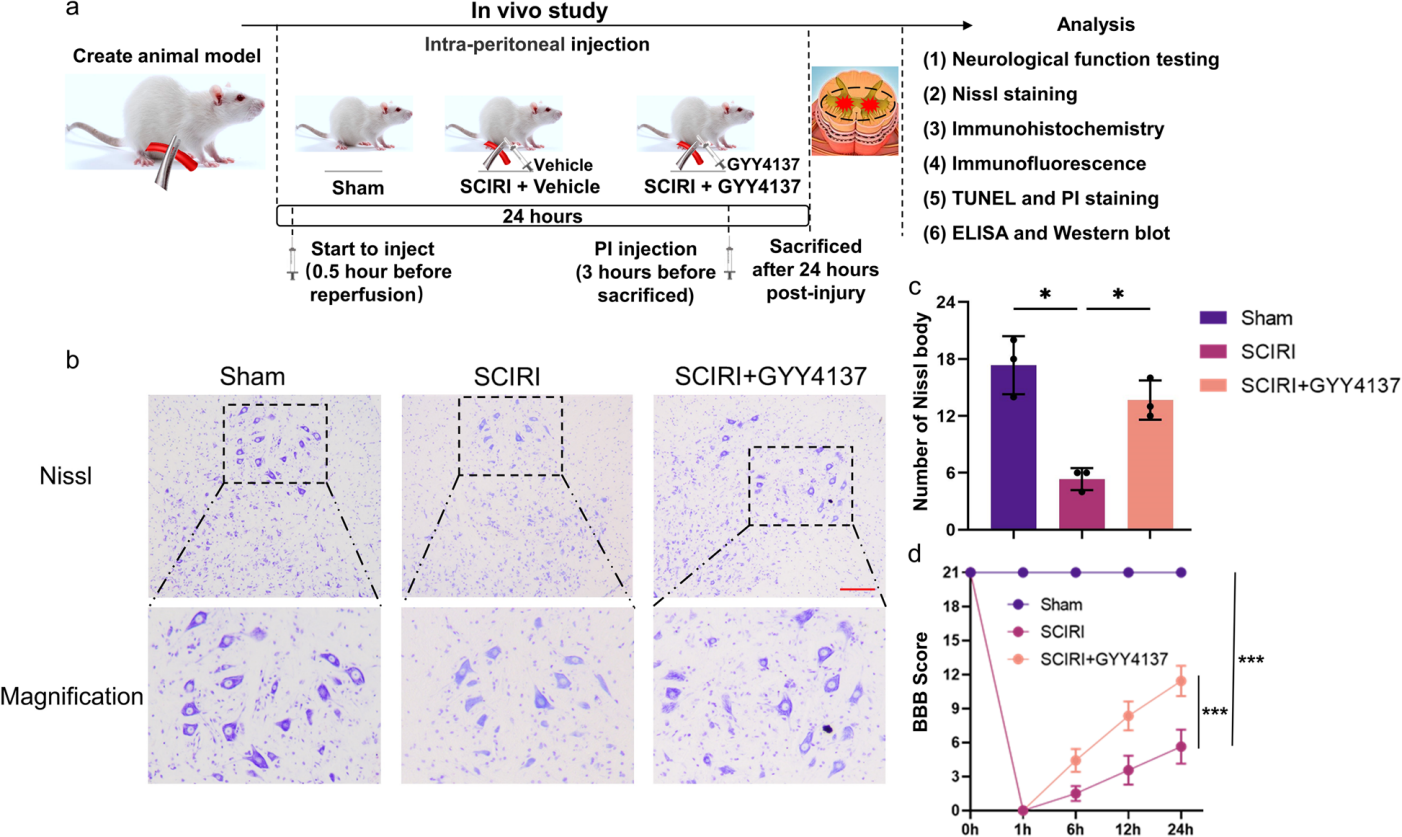
Experimental protocol and effect of H_2_S on spinal cord structure and lower limb motor function in SCIRI rats. **a** Schematic of the timeline of SCIRI induction, GYY4137 administration, and subsequent analysis. **b** Representative spinal cord cross-section Nissl staining showing the ventral horn regions of the spinal cord (100x, scale bar = 200 μm). **c** Quantitative analyses of Nissl body in the ventral horn regions of the spinal cord (*n* = 3 rats per group). **d** The BBB scores of different groups at each time point (*n* = 14 rats per group). Data are presented as mean ± SD. *p < 0.05; ***p < 0.001. BBB, Basso, Beattie & Bresnahan locomotor rating scale; PI, propidium iodide; SCIRI, spinal cord ischemia‒reperfusion injury; TUNEL, terminal deoxynucleotidyl transferase dUTP nick end labeling

A total of 42 rats were divided into three groups (*n* = 14 per group). (a) The sham group (*n* = 14) underwent the surgical procedure without aortic clipping. (b) The SCIRI group (*n* = 14) underwent abdominal aortic exposure and cross-clamping for 60 min followed by intraperitoneal injection of an equivalent volume of vehicle solution immediately after reperfusion. The rats in the SCIRI + GYY4137 group (*n* = 14) underwent the same surgical procedure as the SCIRI group, but were treated with GYY4137 (50 mg/kg) 30 min before the onset of spinal cord reperfusion [27, 29]. Rats were sacrificed for subsequent experiments as described in Fig. 1a.

### Neurological function assessment

Locomotor recovery after SCIRI was assessed using the Basso, Beattie, and Bresnahan (BBB) open-field locomotor scale [30] ranging from 0 (complete paralysis) to 21 (normal locomotion). The BBB scores were recorded at 1, 6, 12, and 24 h during the acute phase after reperfusion by two experienced investigators who were blinded to the experimental design. Disagreements were solved through discussion to reach a consensus.

### Immunofluorescence staining for NeuN, IBA1, cleaved caspase-3, RIP3, NLRP3 and IL-1β

Immunofluorescence staining was performed according to our previous report [31]. Briefly, frozen sections were washed with PBS, washed with PBS containing 0.1% Tween-20 (Beyotime, ST825), and then blocked with 5% bovine serum albumin (BSA, Sigma, A7906) for 30 min at room temperature. The sections were incubated in permeabilization solution (1% Triton X-100, Beyotime, P0096) for 15 min at room temperature and then incubated in primary antibody diluted in primary antibody dilution buffer (Beyotime, P0277) overnight at 4 °C. After rinsing with PBS, the sections were incubated with the corresponding Alexa Fluor-labeled secondary antibody. The sections were mounted with ProLong Gold antifade reagent with DAPI to label the nuclei (Invitrogen, P36935). Traced sections were examined with a Zeiss microscope (Axioscope 5), and the colocalization of proteins was quantified using ZEN Lite Viewer and ImageJ software. The antibodies used were as follows: mouse anti-NeuN (1:400; Cell Signaling Technology; 94403), mouse anti-Iba1 (1:100; Abcam; ab254360), rabbit anti-cleaved caspase 3 (1:400; Cell Signaling Technology; 9664), rabbit anti-RIP3 (1:100; Zenbio; 505431), rabbit anti-NLRP3 (1:100; Hubio; ET1610-93), rabbit IL-1β (1:100; Abcam; ab254360), Alexa Fluor 488-labeled goat anti-rabbit IgG (H + L) (1:400; Beyotime; A0423), Alexa Fluor 488-labeled goat anti-mouse IgG (H + L) (1:400; Beyotime; A0428), Alexa Fluor 555-labeled donkey anti-rabbit IgG (H + L) (1:400; Beyotime; A0453), and Alexa Fluor 555-labeled donkey anti-mouse IgG (H + L) (1:400; Beyotime; A0460).

### Propidium iodide (PI) and terminal deoxynucleotidyl transferase-mediated dUTP nick-end labeling (TUNEL) staining

PI staining was used to label necroptotic cells as previously reported [19]. Briefly, 5 mg/kg PI (Sigma, P4864) was injected intraperitoneally 21 h after SCIRI induction, and then the spinal cord (L1-2) was enucleated for cryosections 24 h after SCIRI induction.

An in-situ cell death detection kit (TUNEL, Roche, 12156792910) was used to detect apoptotic cells according to the manufacturer’s instructions. Briefly, the frozen sections were washed in PBS and then incubated in permeabilization buffer and blocked. Next, TUNEL reaction mixture was applied, and the sections were incubated for 1 h at 37 °C. Finally, sections were mounted with ProLong Gold antifade reagent with DAPI. Traced sections were examined with a Zeiss microscope (Axioscope 5), and staining intensity was quantified using ZEN Lite Viewer and ImageJ software.

### Immunohistochemical staining of Bax, bad and cleaved caspase-8

The staining was performed according to our previous report [27]. Paraffin sections were deparaffinized and rehydrated. Antigen retrieval was performed with improved citrate antigen retrieval solution (Beyotime, P0083) according to the manufacturer’s instructions. Sections were incubated in hydrogen peroxide to quench any endogenous peroxidases, and then blocked with 5% BSA for 30 min at room temperature. Sections were incubated with primary antibodies. An SABC-HRP kit with anti-rabbit IgG (Beyotime, P0615) and an SABC-HRP kit with anti-mouse IgG (Beyotime, P0612) were used according to the manufacturer’s instructions. Positive staining was visualized with a DAB horseradish peroxidase color development kit (Beyotime, P0202). Sections were counterstained with hematoxylin and dipped in acid alcohol as needed before being dehydrated and mounted on coverslips. Traced sections were examined with a Leica microscope (DMIL LED). Immunostaining images and their mean integrated optical densities were quantified using ImageView and ImageJ software, respectively. The antibodies used were as follows: rabbit anti-Bad (1:100; Proteintech; 10435-1-AP), rabbit anti-Bax (1:250; Abcam; ab32503), and mouse anti-cleaved caspase 8 (1:100; Yeasen; 31272ES50).

### Histological analysis

Nissl body staining was performed according to the manufacturer’s instructions, and Nissl staining solution (Beyotime, C0117) was used to stain the sections. Briefly, paraffin sections were deparaffinized and rehydrated. Then, the sections were stained with Nissl staining solution and bleached using 95% ethanol. Subsequently, sections underwent 95% ethanol dehydration, xylene transparency, and neutral gum mounting. Traced sections were examined with a Leica microscope. Immunostaining images and their colocalization were quantified using ImageView and ImageJ software, respectively.

### Western blotting

Western blot analysis was performed as previously described [27]. Briefly, the spinal cords were freshly dissected, homogenized in a RIPA (Beyotime, P0013C) with a protease and phosphatase inhibitor cocktail for mammalian cell and tissue extracts (Beyotime, P1050), and centrifuged in a microcentrifuge. The concentrations of protein samples were determined using a BCA protein assay kit (Beyotime, P0012). Aliquots of protein (50 µg/lane) were fractionated using sodium dodecyl sulfate‒polyacrylamide gel electrophoresis. Subsequently, the samples were transferred to polyvinylidene difluoride filter membranes (0.2 μm, Millipore, ISEQ00010). Afterward, the membranes were blocked, and then probed with different primary antibodies. Subsequently, the membranes were incubated with corresponding secondary antibodies. The chemiluminescence results were recorded using an imaging system (FUSION SOLO S, VILBER). Signal intensities were quantified using ImageJ software. The antibodies used were as follows: rabbit anti-caspase 8 (1:1000; Boster; A00042-3), mouse anti-caspase 8/p43/p18 (1:1000; Proteintech; 66093-1-Ig), rabbit anti-cleaved caspase 7 (1:1000; Cell Signaling Technology; 9491), rabbit anti-caspase 3 (1:1000; Affinity; AF6311), rabbit anti-cleaved caspase 3 (1:1000; Cell Signaling Technology, 9664), rabbit anti-BCL2 (1:1000; Abcam; ab194583), rabbit anti-Bad (1:1000; Proteintech; 10435-1-AP), rabbit anti-Bax (1:1000; Abcam; ab32503), rabbit anti-RIP3 (1:1000; Zenbio; 505431), rabbit anti-phospho-RIP3 (1:1000; Affinity; AF7443), rabbit anti-NLRP3 (1:1000; Hubio; ET1610-93), rabbit anti-RIP1 (1: 1000; Abcam; ab300617), rabbit anti-phospho-RIP1 (1:1000; Affinity; AF7088), mouse anti-MLKL (1:5000; Proteintech; 66675-1-Ig), rabbit anti-phospho-MLKL (1:1000; Affinity; AF7420), rabbit anti-caspase 1 (1:1000; Boster; BM4291), rabbit anti-cleaved caspase 1 (1:1000; Yeasen; 31029ES50), rabbit anti-GSDMD (1:1000; Proteintech; 20770-1-AP), rabbit anti-GSDMD N-Terminal (1:1000; Affinity; DF13758), mouse anti-β-actin (1:5000; Boster; BM0627), horseradish peroxidase (HRP) Conjugated AffiniPure goat anti-mouse IgG (H + L) (1:5000; Boster; BA1050) and HRP Conjugated AffiniPure goat anti-rabbit IgG (H + L) (1:5000; Boster; BA1054).

### Measurement of interleukin-1β (IL-1β) and IL-18

The production of IL-1β and IL-18 was measured in the spinal cord lysates using a commercial IL-1β Cell Lysates Rat ELISA Kit (Invitrogen, ERIL1B) and Rat IL-18 ELISA Kit (Solarbio, SEKR-0054), respectively. The results were normalized to the protein contents of the samples by constructing a standard curve.

### Statistical analysis

Two independent, blinded investigators performed all counting and measurements in duplicate. All data are presented as the mean ± standard deviation (mean ± SD). Analyses of multiple groups was performed using one-way or two-way ANOVA with Dunnett’s multiple comparisons test in GraphPad Prism version 9. For all statistical tests, a *P* value < 0.05 was considered statistically significant.

## Results

### H_2_S ameliorated the loss of motor neurons and lower limb motor dysfunction following SCIRI

We performed Nissl staining to investigate neuronal changes (Fig. 1b). The number of Nissl bodies in the ventral horn of the spinal cord was significantly decreased in the SCIRI group compared with that of the sham group, but increased by GYY4137 treatment (Fig. 1c).

Furthermore, to explore whether H_2_S could ameliorate motor dysfunction induced by SCIRI, we investigated the motor functions of rats with BBB scores. These results were in accordance with the Nissl staining (Fig. 1d). The trends in the different groups demonstrated that motor function was largely decreased in the first few hours after reperfusion, except in rats from the sham group. The SCIRI + GYY4137 group showed greater scores than the SCIRI group in 6 h, 12 and 24 h. Together, these results suggest that H_2_S could ameliorate neurological function deterioration and loss of neurons induced by SCIRI.

### H_2_S diminished the number of TUNEL-positive neurons in the spinal cord

**Fig. 2:**
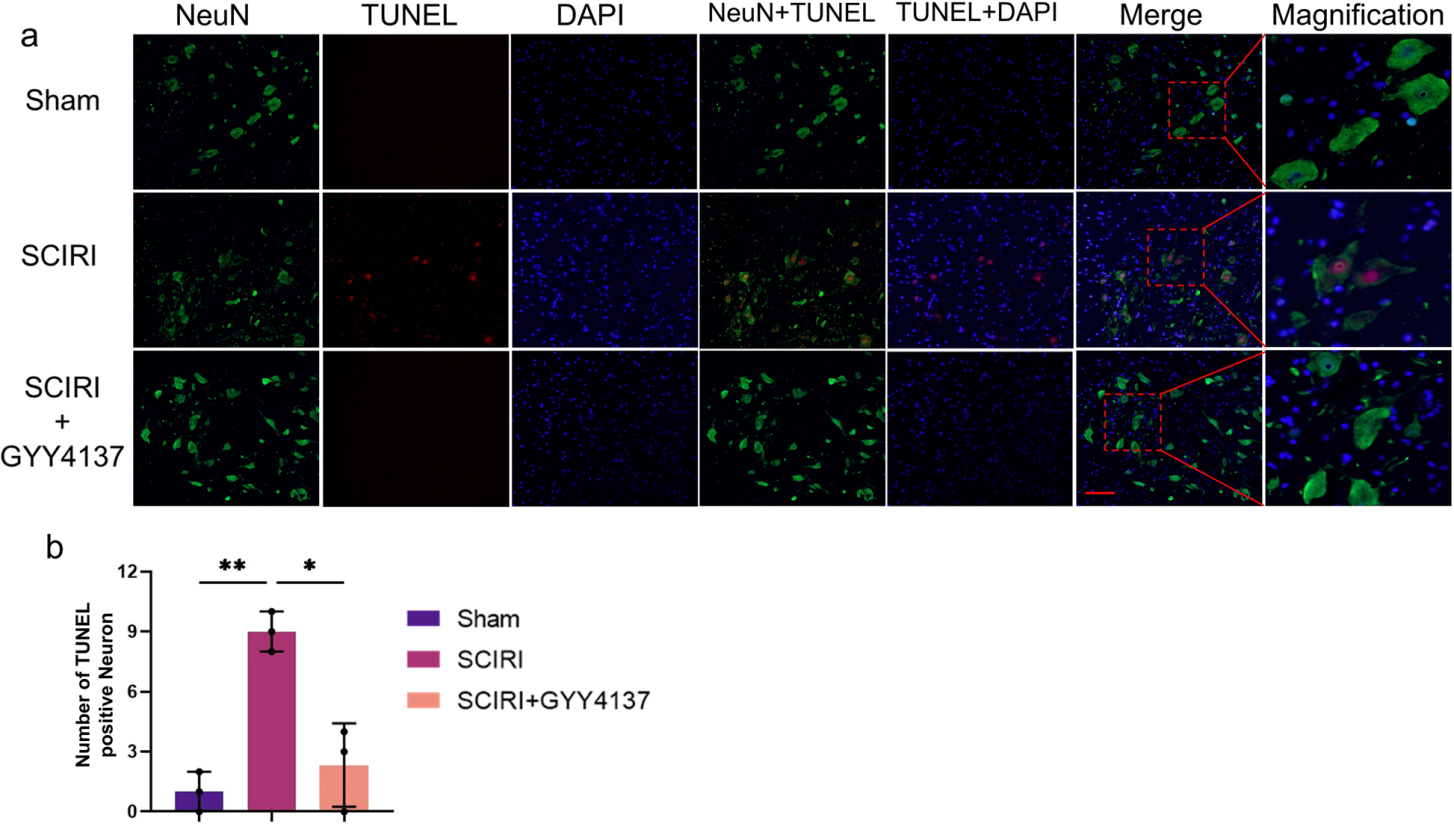
Effect of H_2_S on spinal cord neuron apoptosis in SCIRI rats. **a** Representative images of spinal cord frozen sections labeled with NeuN (green), TUNEL (red) and DAPI (blue) in each group (200x, scale bar = 100 μm). **b** Quantification of TUNEL-positive Neuron counts in the ventral horn regions of the spinal cord (*n* = 3 rats per group). Data are presented as mean ± SD. *p < 0.05; **p < 0.01. DAPI, 4′,6-diamidino-2-phenylindole; NeuN, Neuronal nuclei

We performed colocalization of TUNEL staining, which labels DNA fragmentation generated during apoptosis [32] and NeuN expression, which is a neuron marker [33] to investigate neuronal changes (Fig. 2a). The number of TUNEL-positive neurons was notably increased after SCIRI compared with the sham rats, which was significantly decreased by GYY4137 treatment (Fig. 2b). Taken together, these data indicate that H_2_S significantly decreased the number of apoptotic neurons and inhibited neuronal apoptosis induced by SCIRI.

###  H_2_S reduced SCIRI-induced apoptosis

**Fig. 3:**
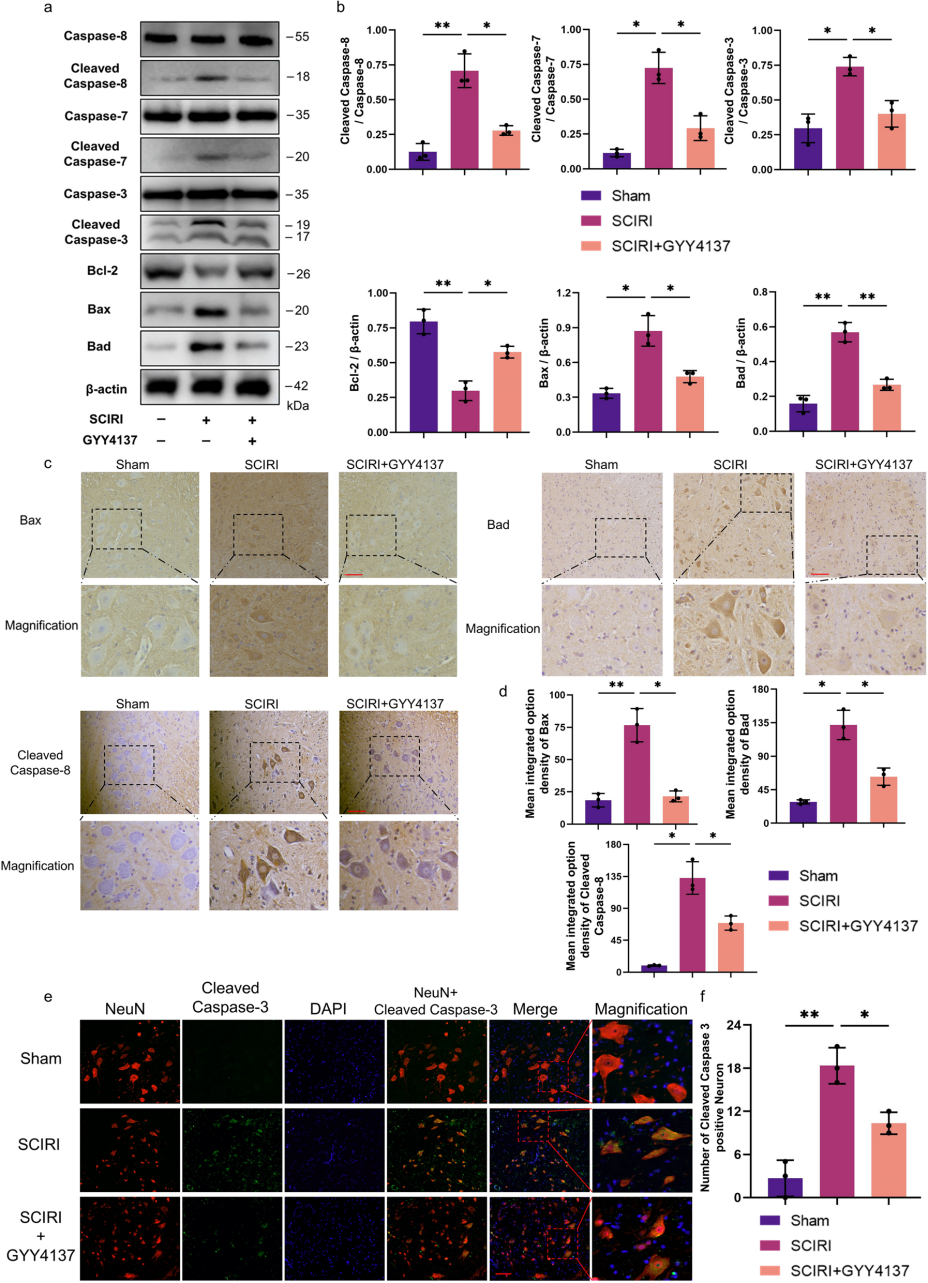
Effect of H_2_S on the expression levels of apoptosis-associated factors in SCIRI rats. **a** The protein levels of caspase-8, cleaved caspase-8, caspase-7, cleaved caspase-7, caspase-3, cleaved caspase-3, Bax, Bcl-2 and Bad were evaluated by Western blotting. β-actin was used to ensure equal loading. **b** Densitometric analysis and quantification of cleaved caspase-8/caspase-8, cleaved caspase-7/caspase-7, cleaved caspase-3/caspase-3, Bax/β-actin, Bcl-2/β-actin, and Bad/β-actin (*n* = 5 rats per group). **c** Representative images of immunohistochemistry staining with Bax, Bad and cleaved caspase-8 in each group (200x, scale bar = 100 μm). **d** Analysis of the mean integrated option density of Bax, Bad and cleaved caspase-8 in each group (*n* = 3 rats per group). **e** Representative image of immunofluorescence staining with NeuN (red), cleaved caspase-3 (green) and DAPI (blue) in each group (200x, scale bar = 100 μm). **f** Quantification of cleaved caspase-3 positive cell counts in the ventral horn regions of the spinal cord (*n* = 3 rats per group). Data are shown as mean ± SD. *p < 0.05; **p < 0.01. Bax, BCL2-associated X protein; Bad, BCL2-associated agonist of cell death

We performed Western blotting to investigate the expression of caspases family members and of B-cell lymphoma 2 (Bcl-2), which are considered key players in regulating apoptosis, to assess the level of apoptosis [17, 31] (Fig. 3a). The Western blotting results demonstrated that expression of the active forms of caspase-3, caspase-7, and caspase-8 were upregulated after SCIRI compared with the level in the sham group, and the cleaved caspase-3/caspase-3, cleaved caspase-7/caspase-7, and cleaved caspase-8/caspase-8 ratios were increased (Fig. 3b). In addition, elevated protein levels of Bax and Bad and decreased protein levels of Bcl-2 were detected in the spinal cords of the SCIRI group (Fig. 3a). With GYY4137 treatment, these changes were largely reversed (Fig. 3b).

Moreover, we performed immunohistochemistry and immunofluorescence colocalization analysis to investigate protein expression and localization. According to the immunohistochemical staining images, Bax, Bad, and cleaved caspase-8 were mainly expressed in the neurons (Fig. 3c). In addition, colocalization of cleaved caspase-3 with NeuN showed that cleaved caspase-3-dependent apoptosis occurred in neurons following SCIRI (Fig. 3e). These results were consistent with the Western blotting results (Fig. 3d, f). Altogether, these results suggest that H_2_S exerted protective effects on SCIRI-induced apoptosis.

###  H_2_S attenuated SCIRI-induced necroptosis

**Fig. 4:**
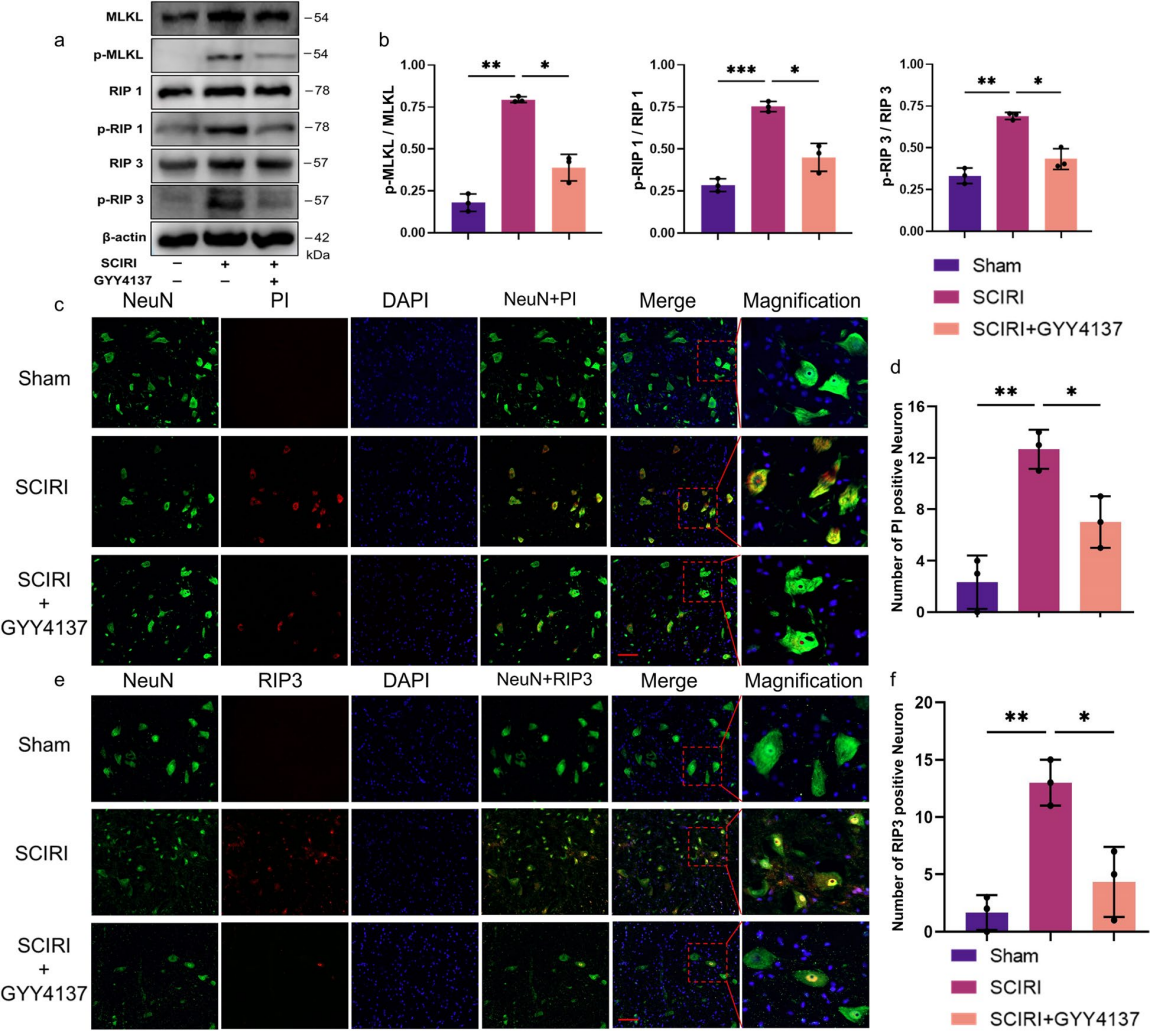
Effect of H_2_S on the expression levels of necroptosis-associated factors in SCIRI Rats. **a** The protein levels of p-MLKL, MLKL, p-RIP1, RIP1, p-RIP3 and RIP3 were evaluated by Western blotting. β-actin was used to ensure equal loading. **b** Densitometric analysis and quantification of p-MLKL/MLKL, p-RIP1/RIP1 and p-RIP3/RIP3 (*n* = 5 rats per group). **c**, **d** Representative image of immunofluorescence staining with NeuN (green), PI (red) or RIP3 (red) and DAPI (blue) in each group, respectively (200x, scale bar = 50 μm). **e**, **f** Quantification of PI-positive or RIP3-positive cell counts in the ventral horn regions of the spinal cord, respectively (*n* = 3 rats per group). Data are shown as mean ± SD. *p < 0.05; **p < 0.01; ***p < 0.001. p-MLKL, Phospho-mixed lineage kinase domain-like protein; p-RIP1/3, Phospho-receptor-interacting protein 1/3

We performed the Western blotting to investigate expression of the necroptosis-associated proteins [34], which are vital proteins for necroptosis activation and include mixed kinase domain-like protein (MLKL), receptor-interacting protein 1 (RIP1), and receptor-interacting protein 3 (RIP3), to assess degree of necroptosis. The Western blotting results demonstrated that expression of the Phospho-MLKL (p-MLKL), p-RIP1, and p-RIP3 in the SCIRI group were higher than those in the sham group (Fig. 4a), and the p-MLKL/MLKL, p-RIP1/RIP1, and p-RIP3/RIP3 ratios were increased (Fig. 4b). With GYY4137 treatment, these changes were largely reversed (Fig. 4a, b).

Additionally, we performed immunofluorescence colocalization analysis of PI, which was used to detect necroptotic cells in the early stage [19, 35], and NeuN to investigate whether necroptosis occurs in neurons (Fig. 4c). The results showed that necroptosis occurred in neurons, and the number of PI-positive neurons showed a dramatic increase following SCIRI compared to those in the sham group, while GYY4137 treatment significantly decreased the number of necroptotic neurons induced by SCIRI (Fig. 4d). Double immunofluorescence staining for RIP3 and NeuN was also performed to locate the necroptotic cells in the neurons (Fig. 4e, f). This result coincided with those of the above PI staining and demonstrated that neuronal necroptosis was involved in SCIRI. Taken together, these results imply that H_2_S effectively attenuated neuronal necroptosis induced by SCIRI.

###  H_2_S attenuated SCIRI-induced pyroptosis, microglia/macrophage activation (M1 polarization) and inflammation

**Fig. 5:**
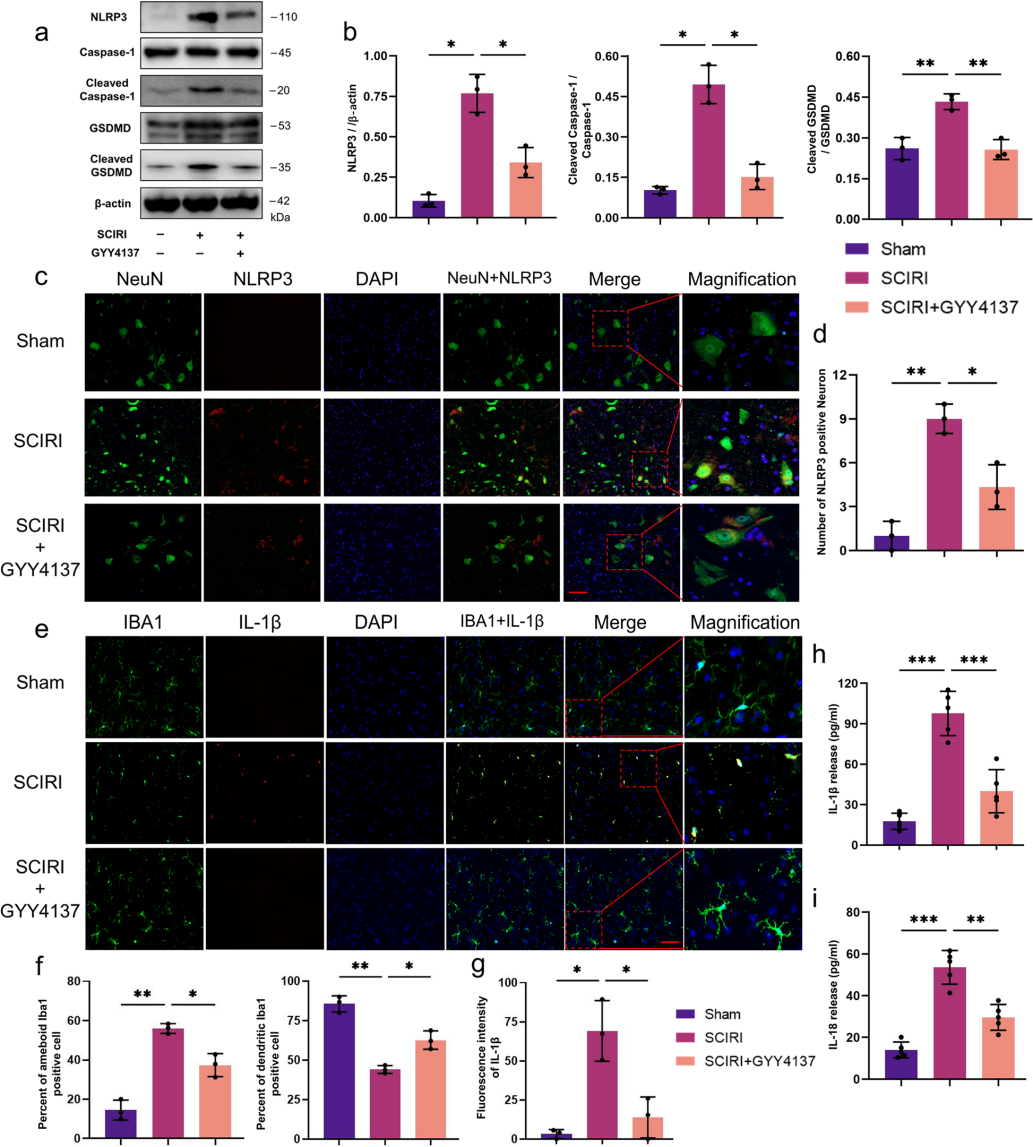
Effect of H_2_S on the expression levels of pyroptosis-associated factors in SCIRI rats. **a** The protein levels of NLRP3, caspase-1, cleaved caspase-1, GSDMD and cleaved GSDMD were evaluated by Western blotting. β-actin was used to ensure equal loading. **b** Densitometric analysis and quantification of NLRP3, cleaved caspase-1 and cleaved GSDMD (*n* = 5 rats per group). **c** Representative image of immunofluorescence staining with NeuN (green), NLRP3 (red) and DAPI (blue) in each group (200x, scale bar = 100 μm). **d** Quantification of NLRP3-positive cell counts in the ventral horn regions of the spinal cord (*n* = 3 rats per group). **e** Representative image of double immunofluorescence staining with IBA1 (green), IL-1β (red) and DAPI (blue) in each group (200x, scale bar = 100 μm). **f** Quantification of different microglial phenotypes in the ventral horn regions of the spinal cord (*n* = 3 rats per group). **g** Quantitative fluorescence intensity of IL-1β (*n* = 3 rats per group). **h**, **i** Quantitative analysis of IL-1β or IL-18 production in spinal cord, respectively (*n* = 5 rats per group). Data are shown as mean ± SD. *p < 0.05; **p < 0.01; ***p < 0.001. GSDMD, gasdermin D; NLRP3, nucleotide-binding oligomerization domain (NOD) -like receptor pyrin domain-containing 3; IL-1β/18, interleukin-1β/18

We performed the Western blotting of canonical molecules of pyroptosis [36], such as NLRP3, cleaved caspase-1 and cleaved gasdermin D (GSDMD), to explore the effects of H_2_S on SCIRI-induced pyroptosis, which has been reported to play a vital role in triggering neuron inflammation (Fig. 5a). The expression levels of NLRP3, cleaved caspase-1, GSDMD and cleaved GSDMD were significantly upregulated after SCIRI and were downregulated after GYY4137 treatment (Fig. 5b). To confirm the locations of pyroptotic cells in the spinal cord, double immunofluorescence staining for NLRP3 and NeuN was performed (Fig. 5c). This result coincided with the Western blotting results and demonstrated that neuronal pyroptosis was involved in SCIRI (Fig. 5d).

Then, colocalization of immunofluorescence staining for Iba1 and IL-1β was performed to investigate microglia/macrophage activation after SCIRI (Fig. 5e). The number of Iba1-positive cells was dramatically increased and the morphology of microglia/macrophages switched from dendritic to activated ameboid in the SCIRI group compared to the sham group. With GYY4137 treatment, the number and morphology of microglia/macrophages remained nearly homeostatic (Fig. 5f). Additionally, the expression level of IL-1β and IL-18 were elevated in the spinal cords of SCIRI rats and reduced after GYY4137 administration (Fig. 5h, i), which was consistent with the immunofluorescence staining results (Fig. 5g). Meanwhile, IL-1β expression was found mainly in Iba1-positive cells (Fig. 5c), indicating that inflammatory cytokines were released by activated microglia/macrophages. More generally, these results suggest that H_2_S plays a protective role in controlling neuronal pyroptosis by inhibiting overactivated microglia-mediated neuroinflammation induced by SCIRI.

## Discussion

The spinal cord, particularly the ventral horn of the spinal cord, is vulnerable to ischemia‒reperfusion insults because the infrarenal aorta is temporarily occluded, resulting in the loss of motor neurons [27]. In this study, we used Nissl staining to verify the reduction in neurons, and BBB scores confirmed lower limb motor function impairment after SCIRI, in line with the results of previous studies [37–39]. The motor functional changes corresponded to the changes in spinal cord structure, in which the number of neurons in the ventral horn was decreased. After treatment with GYY4137, the reduced number of neurons was remarkably reversed, and the number of TUNEL-positive neurons was decreased in SCIRI rats, which implied that H_2_S preserved neurons to a great extent from ischemia‒reperfusion injury.

A considerable number of studies has shown that H_2_S acts as a protective agent via its antiapoptotic and anti-inflammatory properties to counter ischemia-, hypoxia- and oxidative stress-induced neuronal damage [40–42]. However, the effects of H_2_S on PANoptosis in SCIRI remain unclear. The results of this study provide the evidence supporting the anti-PANoptosis effects of H_2_S on SCIRI-induced neuronal death. The major findings are summarized as follows: (a) H_2_S alleviated motor dysfunction and reduced neuron loss after SCIRI; (b) H_2_S prevented SCIRI by inhibiting pyroptosis, apoptosis and necroptosis in neurons; and (c) H_2_S decreased M1 polarization of microglia/macrophages and inflammation after SCIRI.

H_2_S is a gaseous messenger molecule that is produced endogenously from homocysteine, cysteine or 3-mercaptopyruvate and regulates multiple biological functions. It has recently been implicated in various physiological pathological processes in mammals, including cardiovascular disease, acute and chronic inflammation and the function of ion channels [42]. We previously reported that NaHS exhibited protective effects against secondary neuronal injury through the inhibition of malondialdehyde and suppression of reactive oxygen species induced by SCIRI [27]. It causes the release of H_2_S in a spontaneous burst once solubilized, and the effect is short-lived. Therefore, adverse or even toxic reactions are likely to occur [43]. GYY4137, a water-soluble and slow-releasing H_2_S donor, is one of the extremely versatile donor compounds used [43]. It has been reported that GYY4137 could ameliorate the SCIRI-induced neuronal death [44]. The advantage of GYY4137 over sulfide salts, is its ability to release H_2_S in a slow and sustained manner akin to endogenous H_2_S production, rather than that of NaHS as a single concentrated burst. When GYY4137 was administered intraperitoneally to SD rats, the plasma H_2_S concentration increased after 30 min and remained elevated over the next 3 h [45]. In the present study, we injected GYY4137 intraperitoneally to examine the effect of exogenous H_2_S administration and offer a potential therapeutic strategy for SCIRI.

Neuron apoptosis is considered to be a critical process during the pathogenesis of SCIRI, based on the results of our previous studies [17, 31]. The intrinsic and extrinsic pathways are two main pathways of apoptosis [46]. The intrinsic pathway is primarily regulated by Bcl-2 family members, which include antiapoptotic proteins (Bcl-2) and proapoptotic proteins (Bax and Bad). Cytochrome c is triggered to be released into the cytosol by proapoptotic proteins, and it forms the apoptosome, which activates the executioners caspase-3/7 [47]. The extrinsic pathway is governed by death ligands, which can bind death receptors to recruit and activate the critical mediator caspase-8, which either the activates executioner caspase-3 or activates the intrinsic apoptotic pathway [46]. In our present study, the upregulation in expression of cleaved caspase-3/7/8, Bax and Bad, the downregulation of Bcl-2 expression, and the increased number of caspase-3-positive and TUNEL-positive neurons were significantly reduced following SCIRI treatment with GYY4137. Thus, the results of our study confirmed the protective effect of H_2_S against neuronal apoptosis after SCIRI.

Recently, it has been suggested that necroptosis contributes to motor dysfunction after SCIRI [48]. Necroptosis is a nonapoptotic form of PCD that is executed and regulated by programmed mechanisms and mediated by p-RIP1, p-RIP3 and p-MLKL [13]. Necroptotic cells can induce prolonged neurotoxic proinflammatory reactions, which are associated with microglial activation and contribute to neuroinflammation and neuron degeneration [49]. Therefore, we examined the changes in the expression of necroptosis-related molecules. The results showed that GYY4137 treatment inhibited the upregulated expression levels of p-MLKL, p-RIP1, and p-RIP3, along with PI-positive and RIP3-positive necroptotic cells primarily located in neurons in the ventral horn after SCIRI. These results imply that necroptotic neurons are involved in SCIRI, which was rescued by GYY4137 treatment. Thus, H_2_S could exert antinecroptotic effects in neurons after SCIRI.

Neuroinflammation and microglial activation, as noted above, play critical roles in neuron pathological changes after SCIRI [46]. The canonical pyroptotic pathway is mediated by the inflammasome, which includes NOD-like receptor family members, such as NLRP3 and NLRP1, to activate caspase-1, leading to the cleavage of GSDMD and membrane pores on the cell membrane and resulting in membrane rupture [50]. In this study, upregulated expression of NLRP3, cleaved caspase-1, and cleaved GSDMD, as well as elevated IL-1β/18 release, were downregulated by GYY4137 treatment. In addition, double immunofluorescence staining revealed that NLRP3 was expressed within the neuronal population and that IL-1β was expressed by microglia/macrophages. Additionally, the results of this study confirmed that H_2_S prevented neuron pyroptosis, microglia/macrophage activation and inflammation induced by SCIRI.

**Fig. 6:**
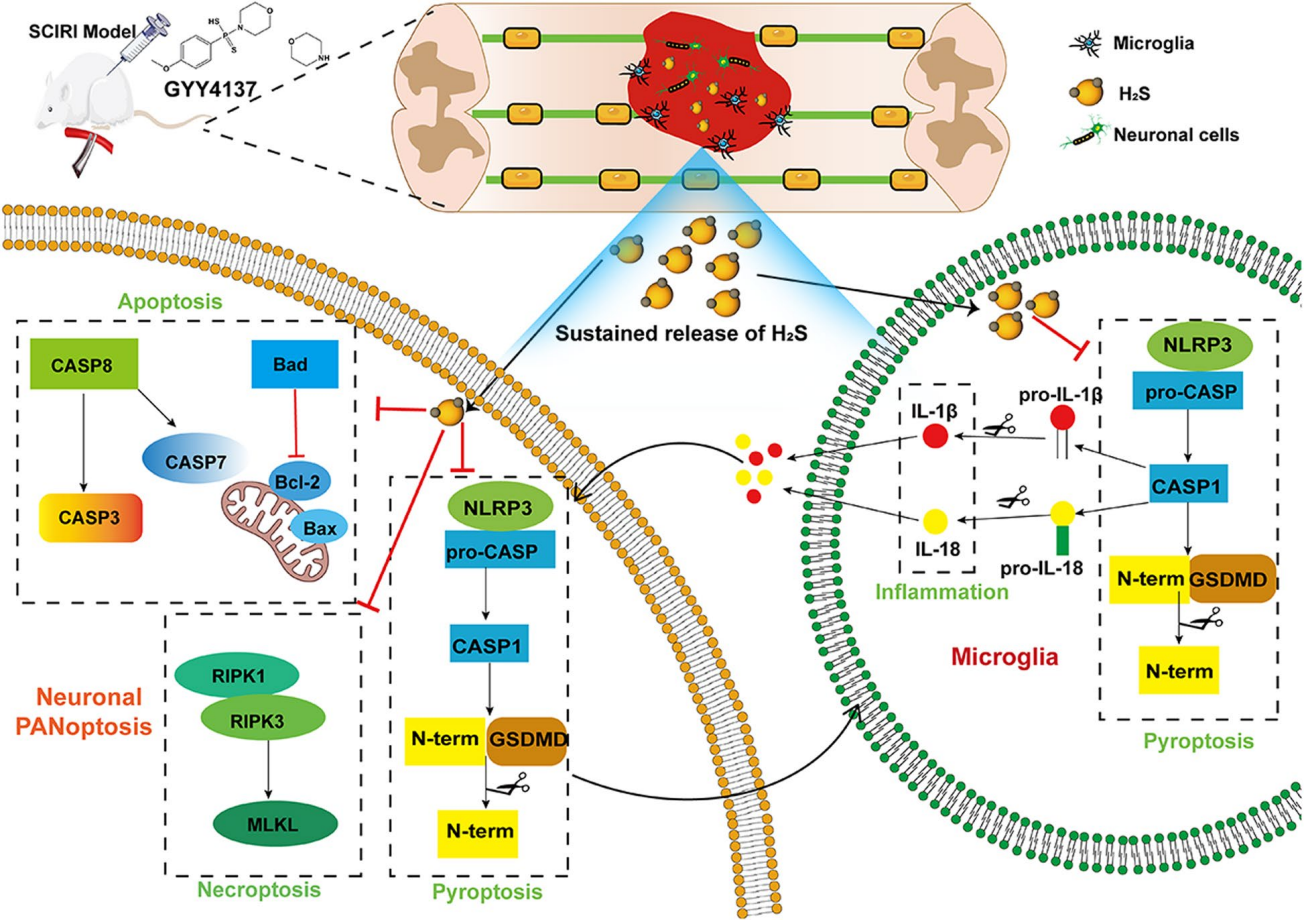
Hypothesized schematic presentation of the protective mechanism of H_2_S in an SCIRI model. H_2_S decreases overactivated microglia-mediated neuroinflammation, inhibits neuronal pyroptosis, apoptosis, and necroptosis (PANoptosis) induced by SCIRI, and promotes neuronal survival

It has been reported that the apoptotic executioner’s caspase-3 and caspase-7 inactivate pyroptosis by cleaving GSDMD at different loci from the inflammatory caspases [51]. Similar to caspase-1, a pyroptotic molecule that can regulate apoptosis, caspase-8, an apoptotic initiator, can also regulate pyroptosis activation [52]. By MLKL-mediated NLRP3 and caspase-1 activation, necroptosis can trigger pyroptosis [53]. Furthermore, caspase-8 acts as a switch to determine the cellular fate toward survival signaling, apoptosis, or necroptosis [52]. Our results and those of other researchers make it clear that cross-talk among the three cell death pathways (pyroptosis, apoptosis, and necroptosis) may be involved in SCIRI, and it is implied that PANoptosis contributes to SCIRI. Many studies have shown that H_2_S affects various diseases [43]; however, few studies have uncovered the anti-PANoptosis properties of H_2_S. The results of our study showed that H_2_S plays multiple regulatory molecular roles and acts as an antiapoptotic agent in protecting neurons after SCIRI by inhibiting neuronal pyroptosis, apoptosis and necroptosis, microglial/macrophage overactivation and the inflammatory response. (Fig. 6). The results of this study showed that H_2_S may preserve spinal cord neurons by mediating microglia/macrophage polarization. These results demonstrate that microglia/macrophages are largely involved in cell death signaling pathways. Therefore, further study should be conducted using innovative drug donors and microglia/macrophage-deficient mice to explore the exact mechanism.

## Conclusions

In summary, the results of our present study demonstrated that H_2_S preserved spinal cord neuron loss, prevented motor dysfunction, reduced inflammatory responses and microglial M1 polarization, and exerted neuroprotective effects via inhibition of neuron pyroptosis, apoptosis, and necroptosis in rats with SCIRI. In this study we identified the involvement of PANoptosis and showed the anti-PANoptosis effect of H_2_S in SCIRI, suggesting a potential application for slow-releasing H_2_S donors as a clinical neuroprotective drug and improving the outcome of patients.

### Supplementary Information

**Additional file 1.**

## Data Availability

The data used to support the findings of this study are available from the corresponding author upon reasonable request.

## Abbreviations

SCIRI: Spinal cord ischemia‒reperfusion injury
PANoptosis: Pyroptosis, apoptosis, and necroptosis
H_2_S: Hydrogen sulfide
PCD: Programmed cell death
NOD: Nucleotide-binding oligomerization domain
NLRP3: Nucleotide-binding oligomerization domain-like receptor pyrin domain-containing 3
(P-)RIP (1/3): (Phospho-) receptor-interacting protein (1/3)
GYY4137: Morpholin-4-ium 4 methoxyphenyl (morpholino) phosphinodithioate
PBS: Phosphate-buffered saline
BBB: Basso, Beattie & Bresnahan locomotor rating scale
PI: Propidium iodide
TUNEL: Terminal deoxynucleotidyl transferase-mediated dutp nick end labeling
DAPI: 4′,6-diamidino-2-phenylindole
NeuN: Neuronal nuclei
Bcl2: B-cell lymphoma 2
Bax: BCL2-associated X protein
Bad: BCL2-associated agonist of cell death
(P-)MLKL: (Phospho-) mixed kinase domain-like protein
GSDMD: Gasdermin D
IL-1β/18: Interleukin-1β/18
NaHS: Sodium hydrosulfide
