# Supplementary figures and images for: A slow-releasing donor of hydrogen sulfide inhibits neuronal cell death via anti-PANoptosis in rats with spinal cord ischemia‒reperfusion injury
Source: Cell Commun Signal. 2024 Jan 12;22:33. doi: 10.1186/s12964-023-01457-x (PMC10785475; doi:10.1186/s12964-023-01457-x)

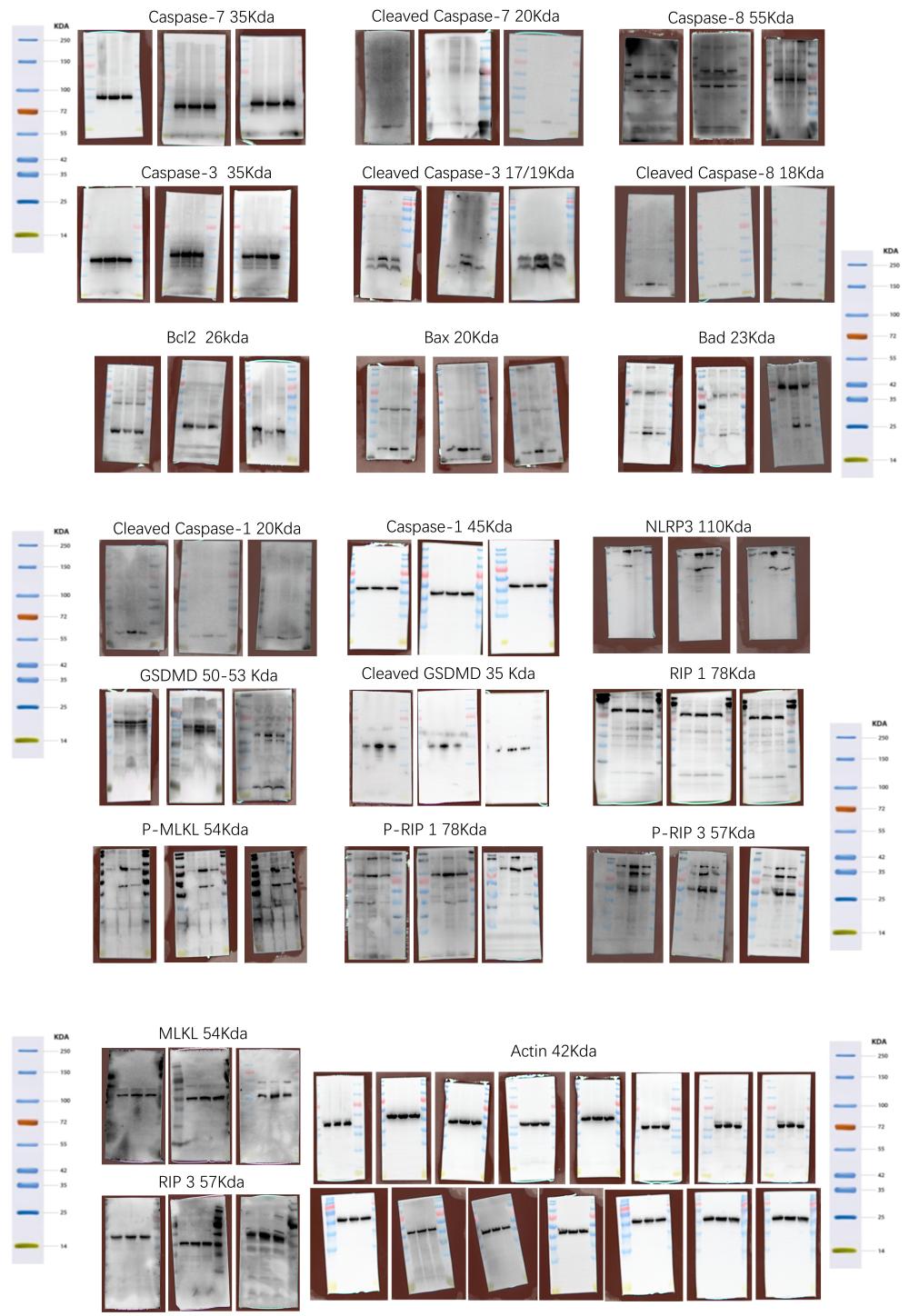

Supplement: Supplementary file 1 — Additional file 1. [file 12964_2023_1457_MOESM1_ESM.jpg]
